# Supplementary material for: Thermodynamic stability of ligand-protected metal nanoclusters
Source: Nat Commun. 2017 Jul 7;8:15988. doi: 10.1038/ncomms15988 (PMC5504301; doi:10.1038/ncomms15988)
Supplement: Supplementary Information [file ncomms15988-s1.pdf]

Type of file: PDF
Title of file for HTML: Supplementary Information
Description: Supplementary Figures, Supplementary Table, Supplementary Notes and Supplementary References.

Type of file: PDF
Title of file for HTML: Peer Review File
Description:

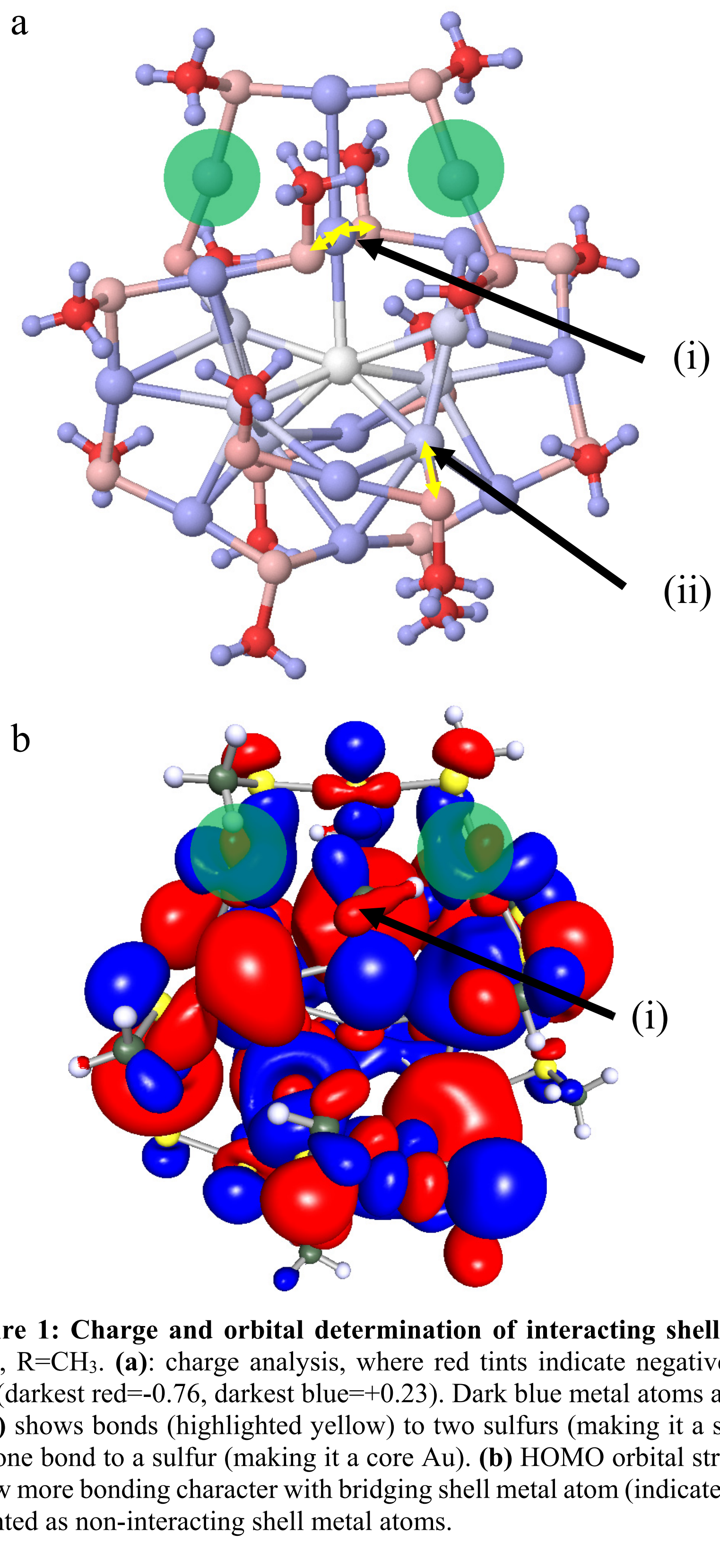

Supplementary Figure 1: Charge and orbital determination of interacting shell atoms. Optimized structure of $Au_{20}SR_{16}$, R=CH_3. **(a)**: charge analysis, where red tints indicate negative and blue positive charges, respectively (darkest red=-0.76, darkest blue=+0.23). Dark blue metal atoms are counted as shell. Similarly, Au atom **(i)** shows bonds (highlighted yellow) to two sulfurs (making it a shell Au), while Au atom **(ii)** only shows one bond to a sulfur (making it a core Au). **(b)** HOMO orbital structure. Highlighted (green) Au atoms show more bonding character with bridging shell metal atom (indicated with black arrow) and therefore are counted as non-interacting shell metal atoms.

Supplementary Note 1:

The metal atoms were first determined as core vs. shell by examining their NBO charge state. Metal atoms with more than 0.2 charge were identified as shell (indicating partially cationic Au atoms). This 0.2 charge threshold was established based on the charge of the shell Au atoms in all of the structures (via same analysis as in Supplementary Fig. 1). For this same core vs. shell determination, alternatively, metal atoms that were coordinated to 2 sulfurs were assigned as shell metal atoms and all other metal atoms were assigned as core. These two methods produced identical results. To determine the number of interacting metal atoms we identified the distance between the shell metal atoms and their nearest core atoms. Assuming an interaction distance cutoff for bonding at approximately 2.5 times the van der wall radii for the Au metals (4 Å), the interacting or non-interacting metals can simply be counted by the number of shell metal atoms with a minimum shell-core distance less or larger than this cutoff. This automated process exactly results in the energy balance shown in Figure 2 for every NC (CE=shell-to-core BE) with the exception of $Au_{20}(SR)_{16}$ (and the negative test $[Cu_{25}(SR)_{18}]^-$ NC). For the $Au_{20}(SR)_{16}$ NC, further examination of the HOMO (electronic) orbital structure indicated primary bonding for 2 of the atoms identified as interacting shell with another shell Au atom as shown in S1. This indicated that these atoms could more accurately be represented as non-interacting shell atoms despite their close proximity to one of the core metal atoms. For the charged systems ($[Au_{25}SR_{18}]^-$, $[Cu_{25}SR_{18}]^-$, and $[Ag_{25}SR_{18}]^-$) we performed vertical electron affinity calculations between the separated core and shell regions to identify where the negative charge will be located. In all cases, the shell region showed a higher electron affinity and the electron was attributed to the shell in the charged systems.

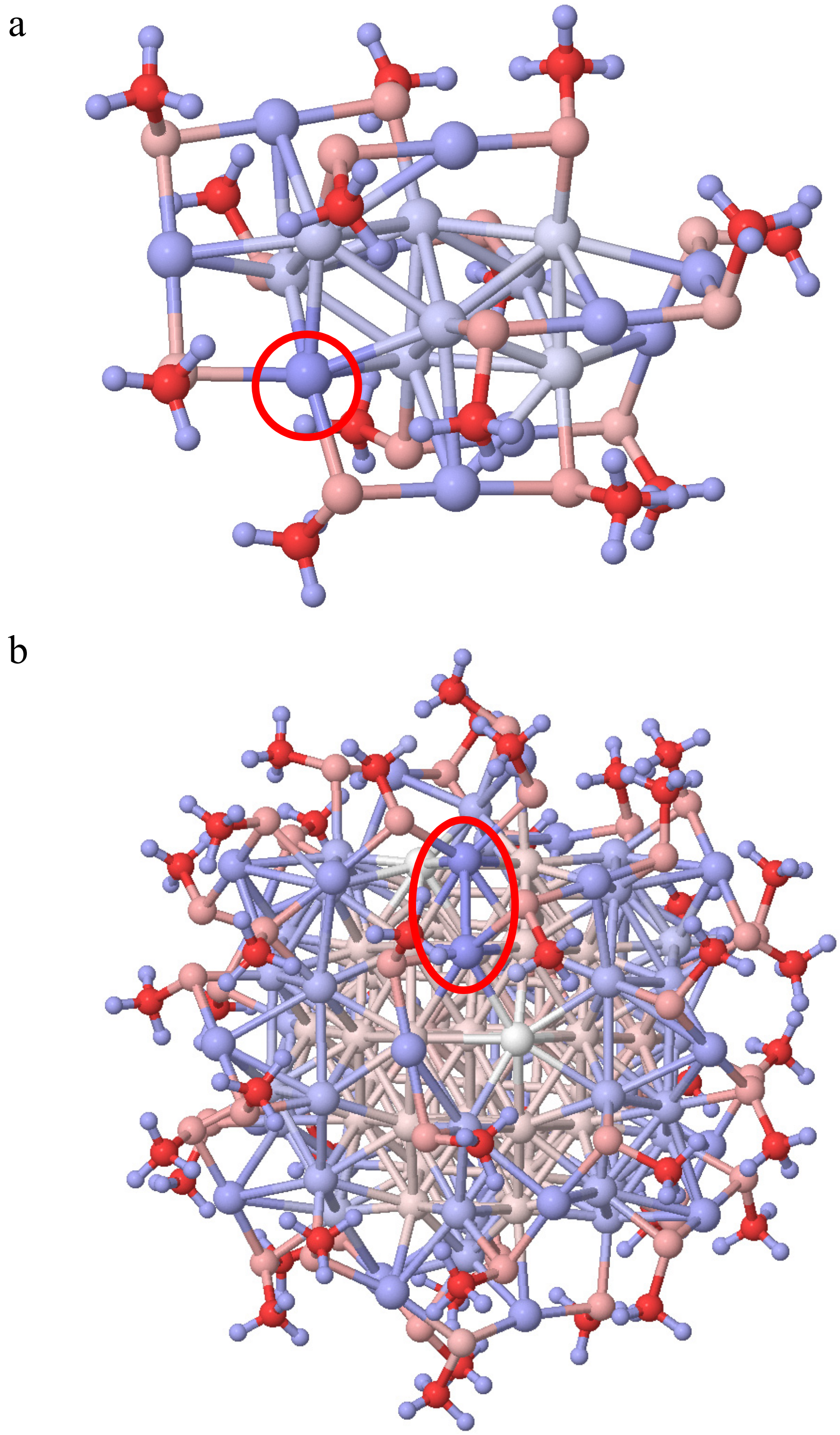

Supplementary Figure 2: Charge analysis highlighting differences between reported core and shell atoms. (a) optimized structure of $Au_{18}SR_{14}$ and **(b)** $Au_{102}SR_{44}$, with R=CH_3. Red tints indicate negative charge (darkest red=-0.76) and blue indicate positive charges (darkest blue=+0.23). Highlighted (with red ovals) Au atoms correspond to atoms previously identified as core[11], while here we show they share the same charge state as the shell Au atoms leading to cores of 8 and 77 Au atoms for $Au_{18}SR_{14}$ and $Au_{102}SR_{44}$,

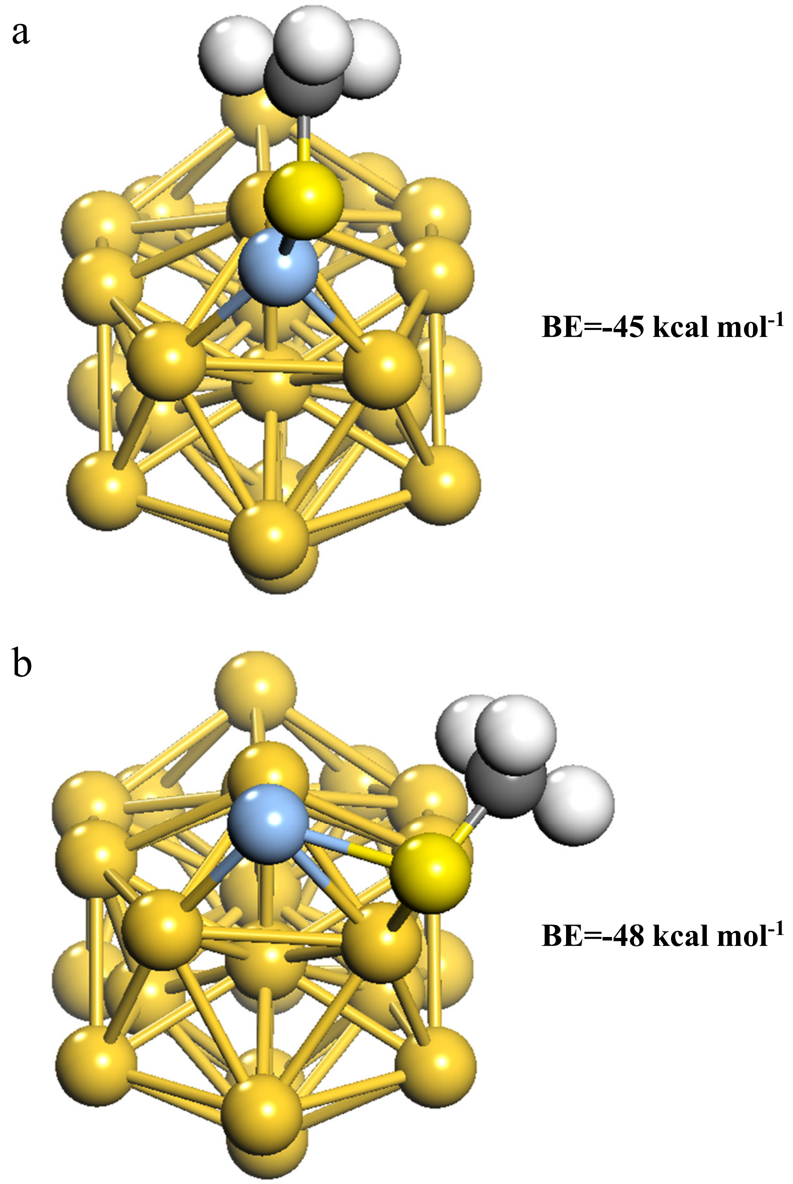

Supplementary Figure 3: Binding energy of sulfur motifs with the core. Optimized structures of a single Au-SR, R=CH_3, ligand placed on the Au_{38} core structure. **(a)** Configuration where the S atom of the ligand interacts with the core metal structure. The shell Au has been colored in blue (the same color scheme as Figure 1) to differentiate from the core Au atoms. **(b)** Configuration where the S atom of the ligand interacts with the core structure.

Supplementary Note 2:

When the sulfur of the ligand if in a non-interacting conformation with the core it showed a BE of -45 kcal mol^{-1}, while the equivalent optimized sulfur-interacting conformation showed a BE of -48 kcal mol^{-1}, indicating the binding between the shell and core is dominated by Au-Au interactions justifying our use of the number of Au atoms as a normalization factor for the shell-to-core BE.

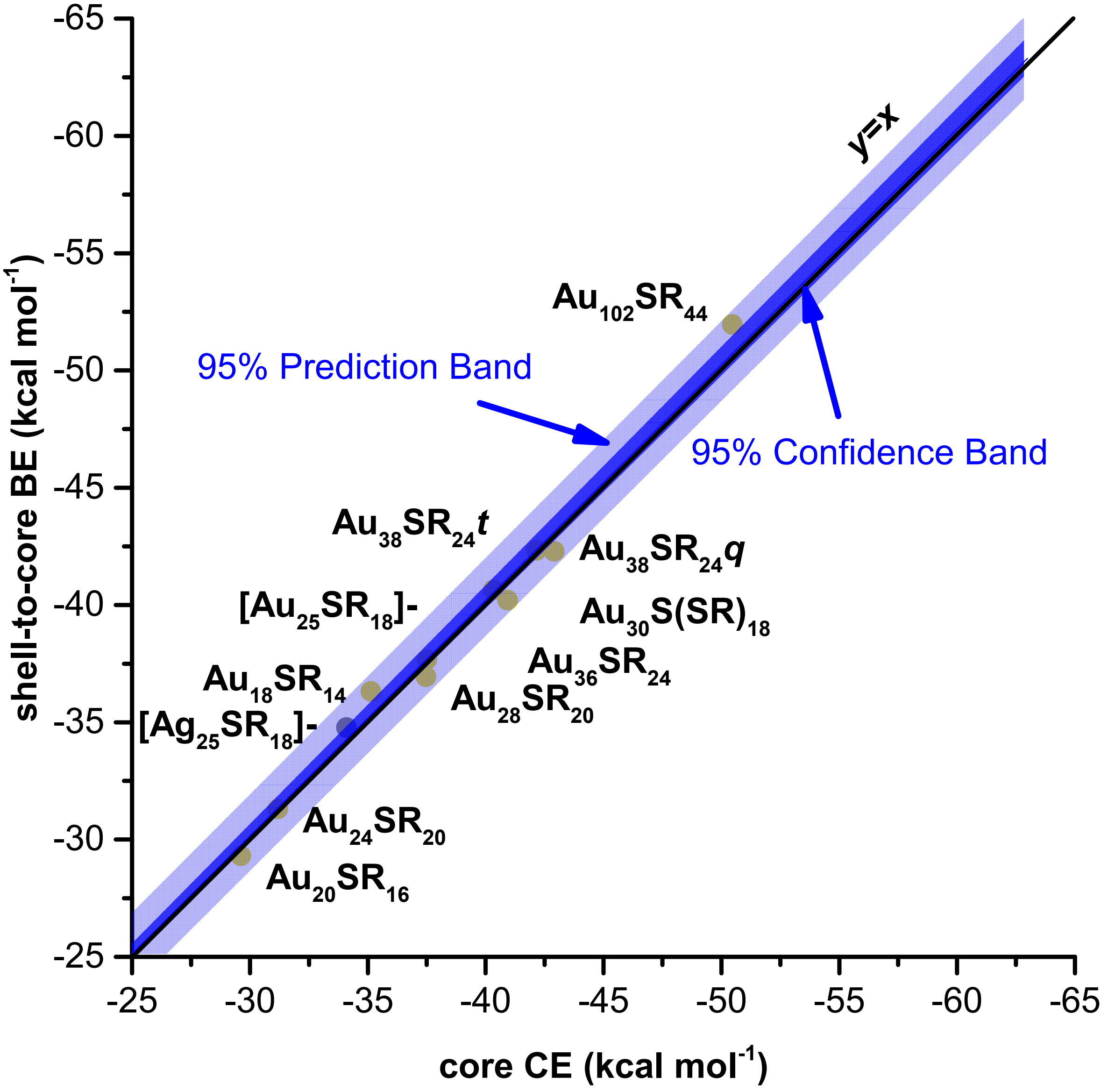

Supplementary Figure 4: Statistical confidence and prediction band definitions. Parity plot between core CE and the shell-to-core BE with only the experimentally-isolated structures. A linear regression along with 95% Confidence and Prediction statistical bands have been superimposed in blue.

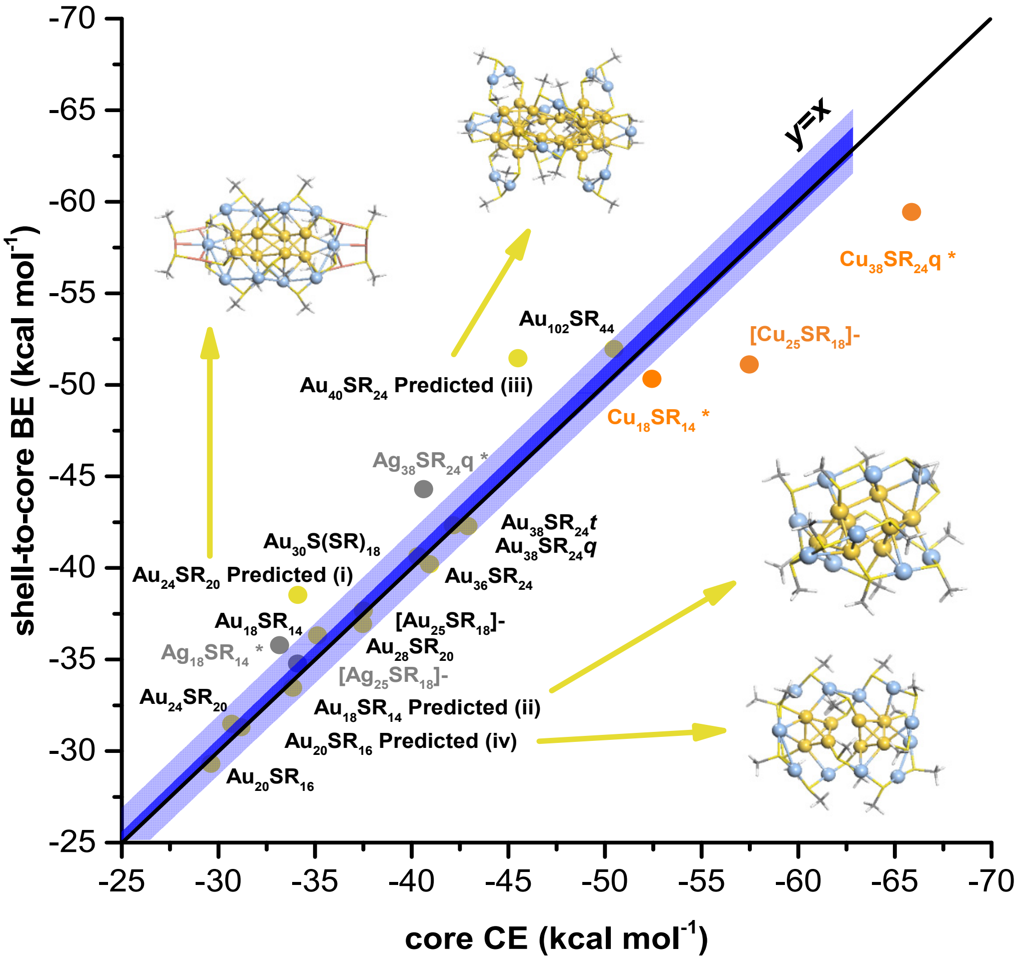

Supplementary Figure 5: Statistical prediction band as stability cutoff. Parity plot between core CE and the shell-to-core BE with identical 95% Confidence and Prediction bands as in Supplementary Fig. 4. Additional points to Figure 2 of the manuscript include: 1) theoretically predicted Au nanoclusters (NCs) **(i)** $Au_{24}SR_{20}$ (ref.[1]), **(ii)** $Au_{18}SR_{14}$ (ref.[2]), **(iii)** $Au_{40}SR_{24}$ (ref.[3]), and **(iv)** $Au_{20}SR_{16}$ (ref.[4]) and 2) NCs of different metals (*) generated and optimized from their analogous experimental Au NC structures.

Supplementary Note 3:

All the experimental NCs fall within the 95% Prediction Bands from the linear regression while all the predicted NCs structures reported, except the $Au_{18}SR_{14}$ and $Au_{20}SR_{16}$ NCs, fall outside the 95% Prediction Bands. We note that both the $Au_{18}SR_{14}$ and $Au_{20}SR_{16}$ predicted NCs showed energetics matching shell-to-core BE and core CE, indicating that these structures fit our thermodynamic stability model but have not yet been experimentally synthesized. Thus, the 95% Prediction bands can be used to distinguish between non-stable and potentially stable NCs.

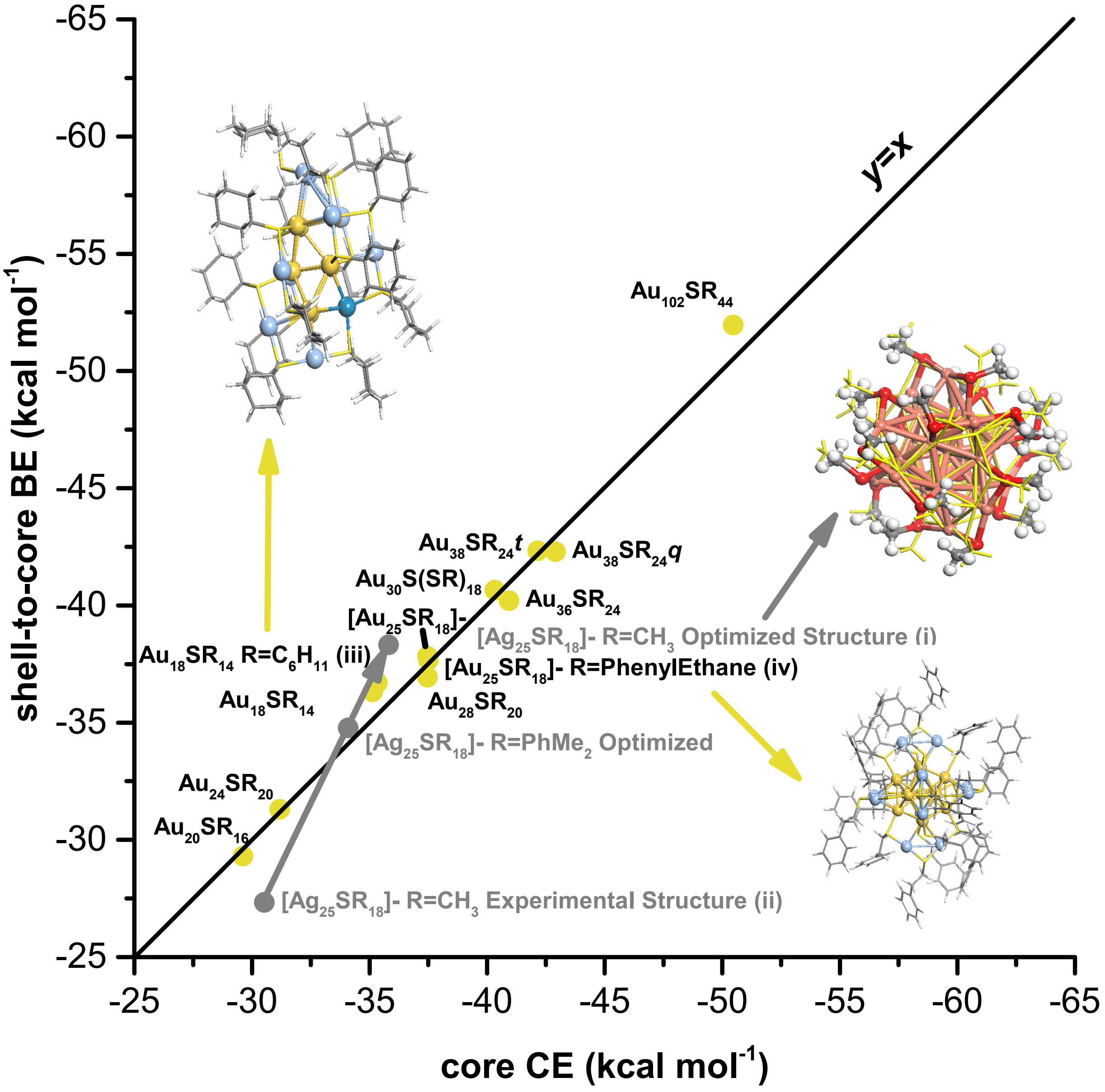

Supplementary Figure 6: Ligand effects in thermodynamic stability theory. Parity plot between core CE and the shell-to-core BE as suggested by our developed thermodynamic stability theory. Most of the values are identical to Figure 2. Additional points include: the $Ag_{25}SR_{18}^-$, with R=CH_3 (**i**) optimized and (**ii**) experimental structures, respectively, (**iii**) the optimized $Au_{18}SR_{14}$ with R=C_6H_{11}, and (**iv**) the $[Au_{25}SR_{18}]^-$, with R=PhenylEthane structures. The silver arrow from **(ii)** to **(i)** indicates the shift of the core CE and shell-to-core BE during optimization of the $[Ag_{25}SR_{18}]^-$ R=CH_3 NC. In the $[Ag_{25}SR_{18}]^-$ R=CH_3 NC image, the red ball/stick represent the experimental structure, whereas, the yellow sticks, the optimized structure.

Supplementary Note 4:

For the $[Ag_{25}SR_{18}]^-$ NC (Supplementary Figs. 6 (i) and (ii)), geometric reconstruction during optimization was noticeable (using as initial state the experimental structure and substituting the R-groups with methyls) and it is due to the lack of hydrogen-bonding in R-groups when R=CH_3. The surface reconstruction of the $[Ag_{25}SR_{18}]^-$ NC with methyls was also evident in the energetics of the NC after optimization when this was the only experimental structure that did not show the BE-CE energy balance. Since we noticed this reconstruction (and CE-BE imbalance), we considered the full ligands and we optimized the experimental $[Ag_{25}(SPhMe_2)_{18}]^-$ NC. Only in this case, we noticed that optimizing the NC accounting for the full ligands results to a perfect CE-BE energy balance. It should be noticed that none of the experimental Au structures showed any similar reconstruction upon methyl substitution and optimization. To further verify that the methyl R-group substitution does not alter the stability of other Au NCs, we optimized the $Au_{18}SR_{14}$ and $[Au_{25}SR_{18}]^-$ NCs with their full ligands, finding variations of only 0.3 kcal mol^{-1} in the core to shell BE while the core CE remained identical to the R=CH_3 structure for each (Figure S6 (iii) and (iv)).

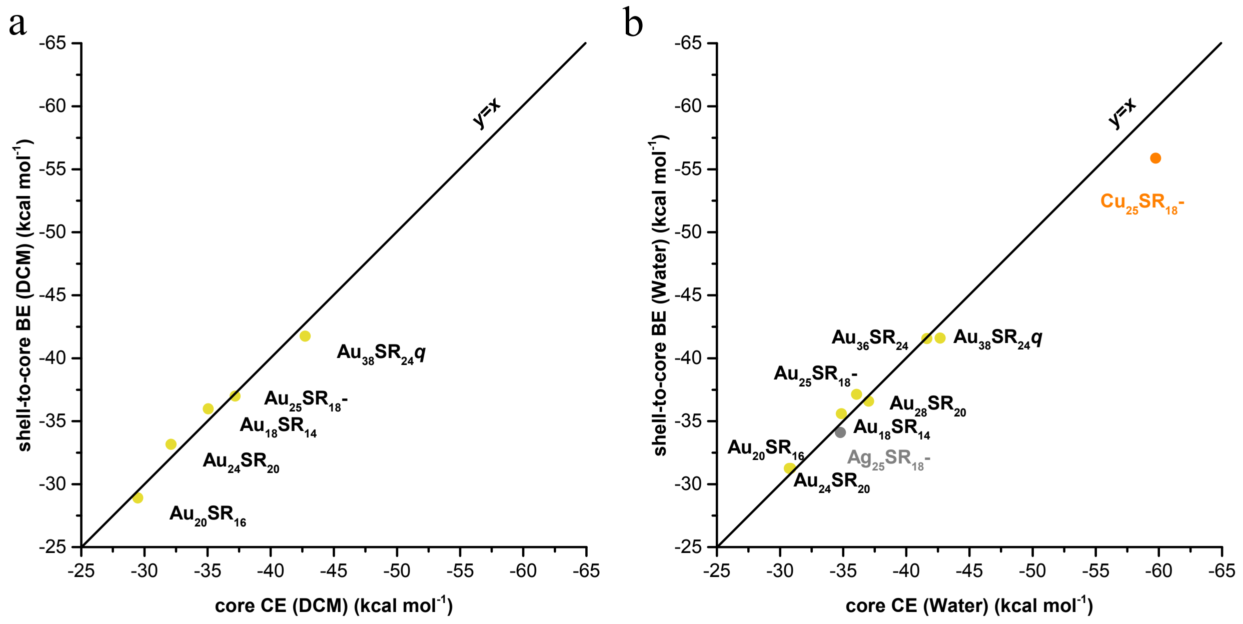

Supplementary Figure 7: Solvent effects in thermodynamic stability theory. Parity plot between core CE (kcal mol^{-1}) and the shell to core BE including **(a)** dichloromethane (**ε**=8.93) and **(b)** water (**ε**=78.46) solvent effects, using the COSMO implicit solvation model.

Supplementary Note 5:

Similar to Figure 2, the parity between shell-to-core BE and core CE holds. Very slight shifts of the shell-to-core BE are introduced by the presence of the solvent without affecting the overall trends.

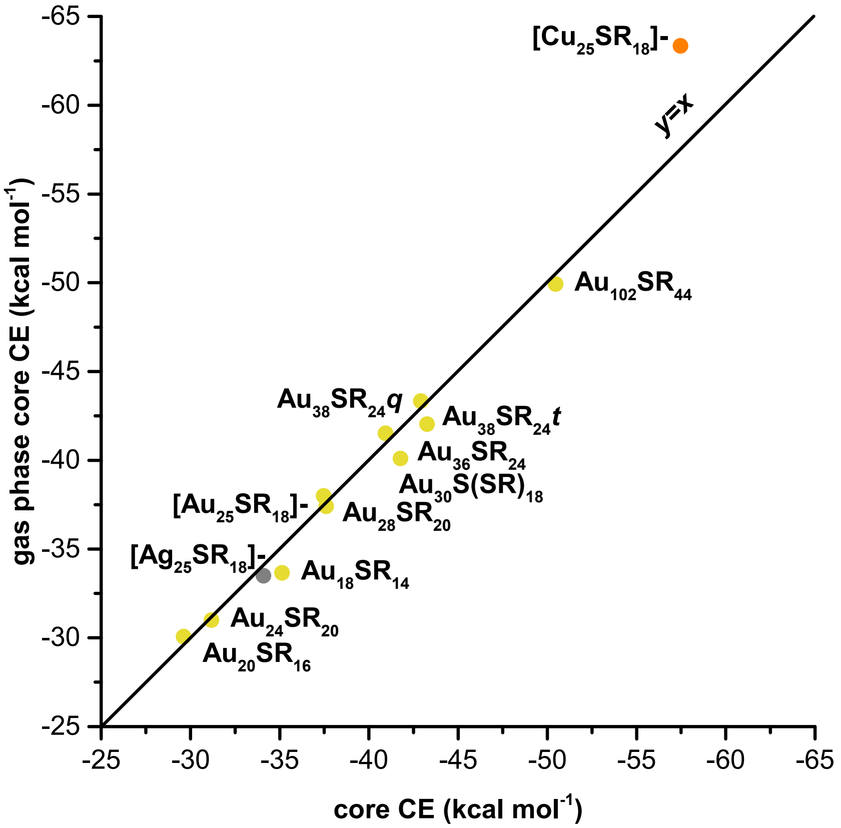

Supplementary Figure 8: Gas phase to core cohesive energy comparison. Parity plot between core CE and the gas phase core CE, indicating remarkable parity between the gas phase and shell-influenced CE values.

Supplementary Table 1: Density Functional comparison.

Method	core CE (kcal mol^{-1})	shell-to-core BE (kcal mol^{-1})
ri-BP-86	-37.82	-37.40
ri-PBE	-38.95	-39.29
ri-BLYP	-31.0	-28.57

Core CE (kcal mol^{-1}) and shell-to-core BE (kcal mol^{-1}) from BP-86[5,6], PBE[7] and BLYP[6,8] from single point energy calculations on the BP-86 optimized $[Au_{25}SR_{18}]^-$ structure. There is a tight match between the core CE and shell-to-core BE for the BLYP, BP-86, and PBE methods, which are all GGA methods.

Supplementary Note 6: Thermodynamic analysis rationalizing the NC stability model.

The stability descriptors of core CE and shell-to-core BE can also be linked to thermodynamic parameters. Since we are separating the NC in two distinct phases (core and shell, according to divide and protect) which are in direct contact, and in order to achieve chemical equilibrium, the partial molar Gibbs free energy (chemical potential (μ)) of the two phases should be equal (so $\Delta\mu$(NC)=0). Towards this end we select a thermodynamic reference state that corresponds to the solution in the Brust-Schiffrin synthesis immediately following the addition of the reducing agent, consisting of solvated M^0 and "staple groups", SR-(M-SR)$_n$, that then, self-assemble to form the NC core and shell regions, respectively. For example, assume a solution where immediately following the addition of the reductant 23 Au^0 atoms exist along with 6 SR-Au-SR-Au-SR and 3 SR-Au-SR groups, in addition to excess thiol and solvent. This would correspond to the initial state of the thermodynamic argument while the final state would be the assembled $Au_{38}SR_{24}$ cluster in the same solution. We can assume that the difference of the partial molar entropy (s) of the M^0 and staple group between the reference solution and the NC phases are equivalent.[9] In addition, the core CE in the presence of the shell can largely represent the partial molar enthalpy (h) of the Au atoms in the core relative to the reference solution phase, as the electronic energy will dominate the h values in a constant volume, liquid phase reaction. To make the analysis of the core CE in the presence of the shell we can rely on the core CE and shell-to-core BE already observed.

$$Core\ CE\ (with\ shell) = \left(E_{Full\ Cluster} - n_c * E_{Metal\ Atom} - E_{ShellInt}\right) \big/ \left(n_c + n_{ShellInt}\right) \quad (1)$$

Where E_X is the electronic energy of species X, n_c is the number of metal atoms in the core, and $n_{ShellInt}$ is the number of interactions between the shell and core. This equation can then be rewritten as:

$$Core\ CE\ (with\ shell) = \left(E_{Full\ Cluster} + n_c * CE_{Core} - E_{Core} - E_{Shell}\right) \big/ \left(n_c + n_{ShellInt}\right) \quad (2)$$

Where $CE_{core}=(E_{core}-n_c*E_{Metal\ Atom})/n_c$, representing the atomization energy for the isolated gas-phase core as defined in the methods section of the manuscript. This equation can then be rearranged as:

$$Core\ CE\ (with\ shell) = \left(n_c * CE_{Core} + n_{ShellInt} * BE_{shell-to-core}\right) \big/ \left(n_c + n_{ShellInt}\right) \quad (3)$$

Where $BE_{shell\text{-}to\text{-}core}=(E_{NC}-E_{core}-E_{shell})/n_{ShellInt}$, as defined in Methods. Thus, the cohesive energy of the metal atoms in the core in the presence of the shell can be viewed as a weighted average of the isolated core CE and shell-to-core BEs. Finally, the shell-to-core BE likewise is treated as the h of the core-binding shell M atoms relative to the reference solution phase, considering interactions

between staple groups are known to be very weak relative to their interactions with the core M^0 atoms.[10] These assumptions are summarized as:

$$\Delta\mu_{M\ Core}^{Solution-NP} = \Delta h_{M\ Core} - T\Delta s_{M\ Core} \approx Core\ CE(with\ shell) - T\Delta s_{M\ Core} \quad (4)$$

$$\Delta\mu_{M\ Shell}^{Solution-NP} = \Delta h_{M\ Shell} - T\Delta s_{M\ Shell} \approx BE_{shell-to-core} - T\Delta s_{M\ Core} \quad (5)$$

Where $\boldsymbol{\mu}$ is chemical potential, h is partial molar enthalpy, s is partial molar entropy, T is temperature, and Solution-NC indicates the difference between the reference solution and NC atoms. Thus, the chemical potential difference between the surface and core metal atoms is given as:

$$\Delta\mu(NP) = 0 = \Delta\mu_{M\ Shell}^{Solution-NP} - \Delta\mu_{M\ Core}^{Solution-NP} \approx BE_{shell-to-core} - Core\ CE\ (with\ shell) \quad (6)$$

Which indicates that for the stable NCs we have identified, this difference in chemical potential will be equal to zero, highlighting a balance of chemical potentials at this core-shell interface. This thermodynamic analysis helps rationalize the lack of temperature-dependence in the stability of NCs in temperature regimes where enthalpic dominate entropic contributions. In addition, it demonstrates why our developed thermodynamic stability model is a valid thermodynamic model for NC stability.

Supplementary References

1. Pei, Y. *et al.* Interlocked catenane-like structure predicted in $Au_{24}(SR)_{20}$: Implication to structural evolution of thiolated gold clusters from homoleptic gold(I) thiolates to core-stacked nanoparticles. *J. Am. Chem. Soc.* **134,** 3015–3024 (2012).

2. Tang, Q. & Jiang, D. E. Revisiting structural models for $Au_{18}(SR)_{14}$. *J. Phys. Chem. C* **119,** 2904–2909 (2015).

3. Malola, S. *et al.* $Au_{40}(SR)_{24}$ Cluster as a Chiral Dimer of 8 □ Electron Superatoms: Structure and Optical Properties. *J. Am. Chem. Soc.* **40,** 26–29 (2012).

4. Pei, Y., Gao, Y., Shao, N. & Xiao, C. Z. Thiolate-protected $Au_{20}(SR)_{16}$ cluster: Prolate Au_8 core with new [$Au_3(SR)_4$] staple motif. *J. Am. Chem. Soc.* **131,** 13619–13621 (2009).

5. Perdew, J. P. Density-functional approximation for the correlation energy of the inhomogeneous electron gas. *Phys. Rev. B* **33,** 8822–8824 (1986).

6. Becke, A. D. Density-functional exchange-energy approximation with correct asymptotic behavior. *Phys. Rev. A* **38,** 3098–3100 (1988).

7. Perdew, J. P., Burke, K. & Ernzerhof, M. Generalized Gradient Approximation Made Simple- ERRATA. *Phys. Rev. Lett.* **77,** 3865–3868 (1996).

8. Lee, C., Yang, W. & Parr, R. G. Development of the Colle-Salvetti correlation-energy formula into a functional of the electron density. *Phys. Rev. B* **37,** 785–789 (1988).

9. Ravi, V., Binz, J. & Rioux, R. Thermodynamic Profiles at the Solvated Inorganic − Organic Interface: The Case of Gold − Thiolate Monolayers. *Nano Lett.* **13,** 4442–4448 (2013).

10. Häkkinen, H. The gold–sulfur interface at the nanoscale. *Nat. Chem.* **4,** 443–455 (2012).

11. Das, A. *et al.* Structure Determination of [$Au_{18}(SR)_{14}$]. *Angew. Chemie Int. Ed.* **54,** 3140–3144 (2015).
